# Retrieval Practice Enhances Lexico-Semantic Representations and Facilitates Word Identification During the Attentional Blink: Evidence from L3 French Compound Word Learning

**DOI:** 10.3390/bs16081438

**Published:** 2026-08-20

**Authors:** Su Liu, Shan Huang, Jiawen Han, Xinyi Wang, Siwei Long, Hong-Wen Cao

**Affiliations:** 1 School of Foreign Languages and Cultures, Chongqing University, Chongqing 400044, China; 2 Research Center for Language Cognition and Language Application, Chongqing University, Chongqing 400044, China

**Keywords:** retrieval practice, attentional blink, rapid serial visual presentation, third language vocabulary learning, lexico-semantic access

## Abstract

The present study investigated whether retrieval practice facilitates memory retention and the rapid identification of newly learned French word components under attentional blink (AB) conditions. Specifically, a total of 107 native Chinese speakers participated in the study, and 103 were included in the final analyses after exclusions. In Experiment 1, participants studied novel, semantically opaque French compound words under two learning conditions: retrieval practice and repeated study. Experiment 2 was conducted 15 min after the completion of Experiment 1. Participants performed a two-target rapid serial visual presentation (RSVP) task in which the morphemic components of the learned compound words were presented as the first target (T1) and the second target (T2) at critical lags (Lag 3 and Lag 7). Experiment 1 confirmed the classical testing effect, with the retrieval practice condition showing higher accuracy and faster response times than the repeated study condition. Experiment 2 revealed that participants in the retrieval practice condition exhibited significantly higher T2 identification accuracy at Lag 7 and a numerical advantage at Lag 3 compared with those in the repeated study condition. These findings indicate that retrieval practice facilitates T2 identification, particularly when attentional resources are relatively recovered at Lag 7. Overall, retrieval-based learning appears to strengthen newly acquired lexico-semantic representations and support the rapid identification of newly learned word components under RSVP conditions.

## 1. Introduction

Retrieving information from memory is widely known to enhance later learning and long-term retention. Compared with repeated study, retrieval practice often produces more durable and accessible memory traces, a phenomenon commonly referred to as retrieval-based learning, the retrieval practice effect, or the testing effect (Adesope et al., 2017; Karpicke, 2017; Karpicke et al., 2014; Karpicke & Roediger, 2008; Rickard & Pan, 2018; Roediger & Butler, 2011; Roediger & Karpicke, 2006; Rowland, 2014; van den Broek et al., 2016; Yang et al., 2021). Several theoretical accounts have been proposed to explain this advantage, including retrieval effort theory, desirable difficulties theory, the distribution-based bifurcation model, and episodic context theory (E. L. Bjork & Bjork, 2011; R. A. Bjork, 1994; R. A. Bjork & Bjork, 1992; Glover, 1989; Halamish & Bjork, 2011; Howard & Kahana, 2002; Karpicke & Roediger, 2007; Kornell et al., 2011; Lehman & Malmberg, 2013; Pyc & Rawson, 2009). Despite their differing emphases, they converge on the view that active retrieval changes the quality of subsequent memory representations, rather than merely providing another opportunity for exposure.

A particularly relevant account for vocabulary learning is the elaborative retrieval theory. Based on the spreading activation model of semantic memory, Carpenter (2009, 2011) proposed that successful retrieval activates information semantically related to the cue and integrates it with the retrieved target. From this perspective, retrieval practice strengthens memory not only by increasing access to the target item, but also by enriching the semantic network surrounding that item (Carpenter & Yeung, 2017; Collins & Quillian, 1972). This view is supported by behavioral and neurocognitive evidence. For example, retrieval practice has been shown to benefit second language vocabulary learning as well as third language (L3) vocabulary learning (Huang et al., 2025; Li et al., 2022; Long et al., 2026; van den Broek et al., 2013), and neuroimaging and electrophysiological studies suggest that retrieval-based learning is associated with more efficient semantic processing and stronger semantic representations (Bridge & Paller, 2012; Li et al., 2023; Rosburg et al., 2015; van den Broek et al., 2013; Wing et al., 2013; Zhuang et al., 2022). These findings indicate that retrieval practice may influence not only later memory performance, but also the way newly learned words are accessed and processed under subsequent task demands.

The attentional blink (AB) provides a useful paradigm for examining rapid visual processing under temporal constraints. In a rapid serial visual presentation (RSVP) stream, participants are typically able to identify the first target (T1) with relatively high accuracy, but their identification of the second target (T2) is impaired when T2 appears approximately 200–500 ms after T1 (Chun & Potter, 1995; Raymond et al., 1992). This transient deficit is generally taken to reflect limitations in temporal attention and working memory consolidation (Dux & Marois, 2009; Martens & Wyble, 2010). According to the episodic Simultaneous Type/Serial Token model, the AB occurs because the cognitive system temporarily suppresses subsequent input while T1 is being consolidated, thereby preserving the episodic distinctiveness of rapidly presented stimuli (Wyble et al., 2009). Therefore, the AB paradigm offers a sensitive way to investigate how rapidly presented information is encoded, consolidated, and disrupted when attentional resources are limited.

Importantly, the magnitude of the AB is not fixed. Previous research suggests that the relationship between T1 and T2 affects T2 identification. When T1 and T2 are semantically or lexically related, T2 is more likely to be detected, even when it appears within the typical AB window (Cao et al., 2014, 2023; Potter et al., 2002). For example, studies using bilingual materials have shown that translation equivalents can facilitate T2 identification, suggesting that processing T1 may pre-activate lexico-semantic information related to T2 (Cao et al., 2023; Thierry & Wu, 2007; Wu & Thierry, 2010). Such findings suggest that semantic activation can partly support T2 identification during RSVP processing. More broadly, they indicate that the AB paradigm can be used to examine how semantic processing interacts with attentional selection during rapid visual word recognition.

However, it remains unclear whether learning history can shape subsequent rapid visual identification of newly learned words. Previous studies on retrieval practice have primarily focused on long-term retention, recognition accuracy, or neural correlates of vocabulary learning. Far less is known about whether retrieval practice, compared with repeated study, facilitates the online identification of newly learned words when they are presented rapidly and under temporal pressure. This question is particularly relevant for L3 vocabulary learning, where newly acquired lexical representations are still fragile and may depend strongly on the learning conditions under which they were encoded.

The present study therefore examined whether retrieval practice, compared with repeated study, facilitates memory retention and subsequent rapid identification of newly learned L3 French word components under RSVP conditions. Participants first learned semantically opaque French compound words under retrieval practice and repeated study conditions. They then completed an RSVP task in which the two morphemic components of each learned compound word were presented as T1 and T2 at Lag 3 and Lag 7. We predicted that recognition memory would be better following retrieval practice than repeated study and that retrieval practice would facilitate T2 identification in the RSVP task. Two possible patterns were considered. If retrieval practice specifically attenuates the attentional blink, the retrieval practice advantage should be stronger at Lag 3 than at Lag 7. Alternatively, if retrieval practice produces a more general benefit by strengthening newly learned lexico-semantic representations, the advantage may emerge more clearly when attentional resources are relatively recovered, without a reliable Learning Condition × Lag interaction.

## 2. Experiment 1

### 2.1. Methods

#### 2.1.1. Participants

A total of 107 Chinese–English bilingual students (50 males; age range = 18–25 years, *M* = 20.02) were recruited from Chongqing University, all of whom were right-handed and had normal or corrected-to-normal vision. None of the participants reported any prior formal experience of learning French. All participants provided written informed consent before taking part in the experiment. Four participants were not included in the final analyses because they did not follow the experimental instructions.

All participants had passed the College English Test Band 4 (CET-4), a national English proficiency test for university students in China, with an average score of 520.13 (*SD* = 47.32; pass mark = 425; maximum score = 710). Their English lexical proficiency was further assessed using LexTALE, which included 20 non-words and 40 words (Lemhöfer & Broersma, 2012). The mean LexTALE score was 57.46 (*SD* = 7.49), suggesting that the sample, on average, had lower-intermediate English proficiency. Participants also rated their English listening (*M* = 3.55, *SD* = 0.57), speaking (*M* = 3.42, *SD* = 0.53), reading (*M* = 3.80, *SD* = 0.48), and writing abilities (*M* = 3.63, *SD* = 0.59) on a five-point scale. Together, these measures suggest that the participants were unbalanced Chinese–English bilinguals with lower-intermediate to intermediate English proficiency. The study was approved by the Ethics Committee of Chongqing University.

#### 2.1.2. Materials

A total of 96 hyphenated French compound words were drawn from the Lexique2 database (New et al., 2004). Among these materials, 24 Chinese–French word pairs were randomly assigned to the repeated study condition, while another 24 pairs were randomly assigned to the retrieval practice condition. The remaining 48 French compound words were not included in the learning phase and served as distractors in the final recognition test. The average frequency of French compound words was obtained using the Lexique2 database (*M* = 2.20, *SE* = 0.13), and the SUBTLEX-logW (Cai & Brysbaert, 2010) database was used to calculate the average frequency of Chinese compound words (*M* = 1.78, *SE* = 0.12).

Paired-sample *t*-tests were conducted to compare the lexical characteristics of the materials assigned to the two learning conditions. *T*-test results for French compound words revealed no significant differences in word frequency (*t*(23) = −0.13, *p* > 0.05) or word length (*t*(23) = −0.81, *p* > 0.05) between the retrieval practice and the repeated study conditions. Similarly, paired-sample *t*-tests for Chinese compound words revealed no significant differences in word frequency (*t*(23) = −0.48, *p* > 0.05) or Chinese character strokes (*t*(23) = 1.26, *p* > 0.05) between the two learning conditions.

#### 2.1.3. Design and Procedure

**Figure 1:**
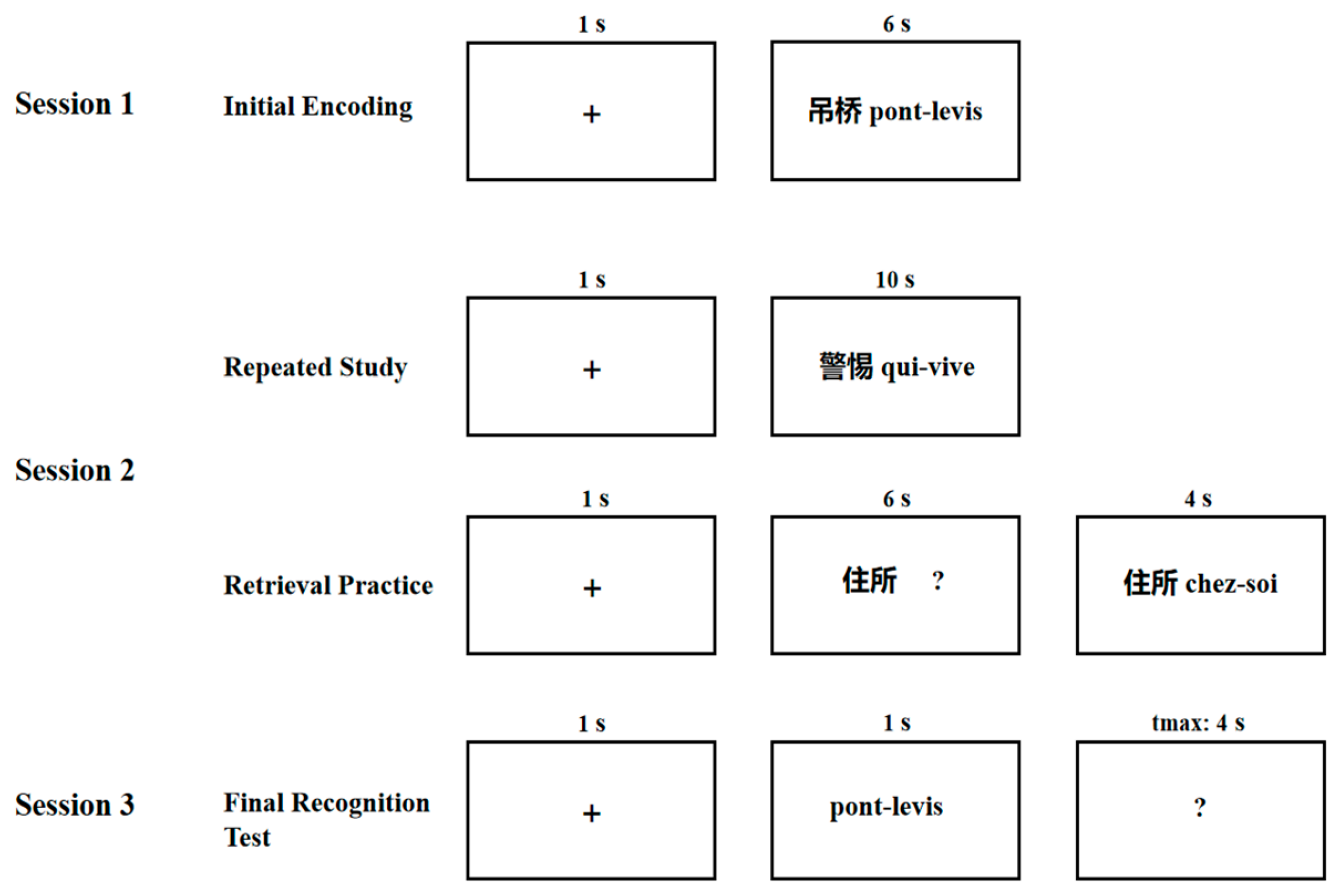
Schematic illustration of the experimental procedure.

Experiment 1 began with an initial encoding session, followed by retrieval practice and repeated study, and ended with a final recognition test, as shown in Figure 1. In the first session, all participants studied 48 French hyphenated compound words together with their Chinese translations. Each trial began with a fixation cross presented for 1 s, followed by a French–Chinese word pair displayed for 6 s. The word pairs were presented individually in randomized order. Participants were instructed to study the pairs carefully. Following the initial encoding phase, participants completed a backward-counting distractor task for 3 min to reduce interference from other memories, repeatedly subtracting three from a randomly selected three-digit number.

Session two was the intervening phase with three blocks. Each block contained 48 Chinese–French word pairs from the initial learning phase, 24 of which were presented in the retrieval practice condition and the remaining 24 in the repeated study condition. The word pairs were randomly displayed in both training conditions, and each experimental stimulus began with a fixation for 1 s. In the repeated study condition, the assigned Chinese–French word pairs were displayed in randomized order for 10 s each. Participants were instructed to study and remember the word pairs silently. In the retrieval practice condition, the 24 Chinese cues were presented individually on the screen’s left side for 6 s (e.g., 住所 + __). During this interval, participants typed the corresponding French hyphenated compound word. After each retrieval attempt, the complete Chinese–French word pair was presented for 4 s as corrective feedback (e.g., 住所 + chez-soi).

Following a filled delay of 30 min, participants completed the final old/new recognition test. The test comprised 48 previously studied French hyphenated compound words (“old”) and 48 unstudied French hyphenated compound words (“new”), which were presented in randomized order at the center of the screen. Each test trial began with a fixation cross displayed for 1 s, followed by a French word presented for 1 s. A question mark then appeared for up to 4 s. Participants were instructed to make an old/new recognition judgment as quickly and accurately as possible by pressing either the “F” or “J” key, with key assignment counterbalanced across participants. The space bar was used when participants were unable to make a judgment. Visual stimuli were displayed on a laboratory computer equipped with a 60 Hz monitor at a resolution of 1920 × 1080 pixels. Participants viewed the screen from a distance of approximately 60 cm.

#### 2.1.4. Results

**Figure 2:**
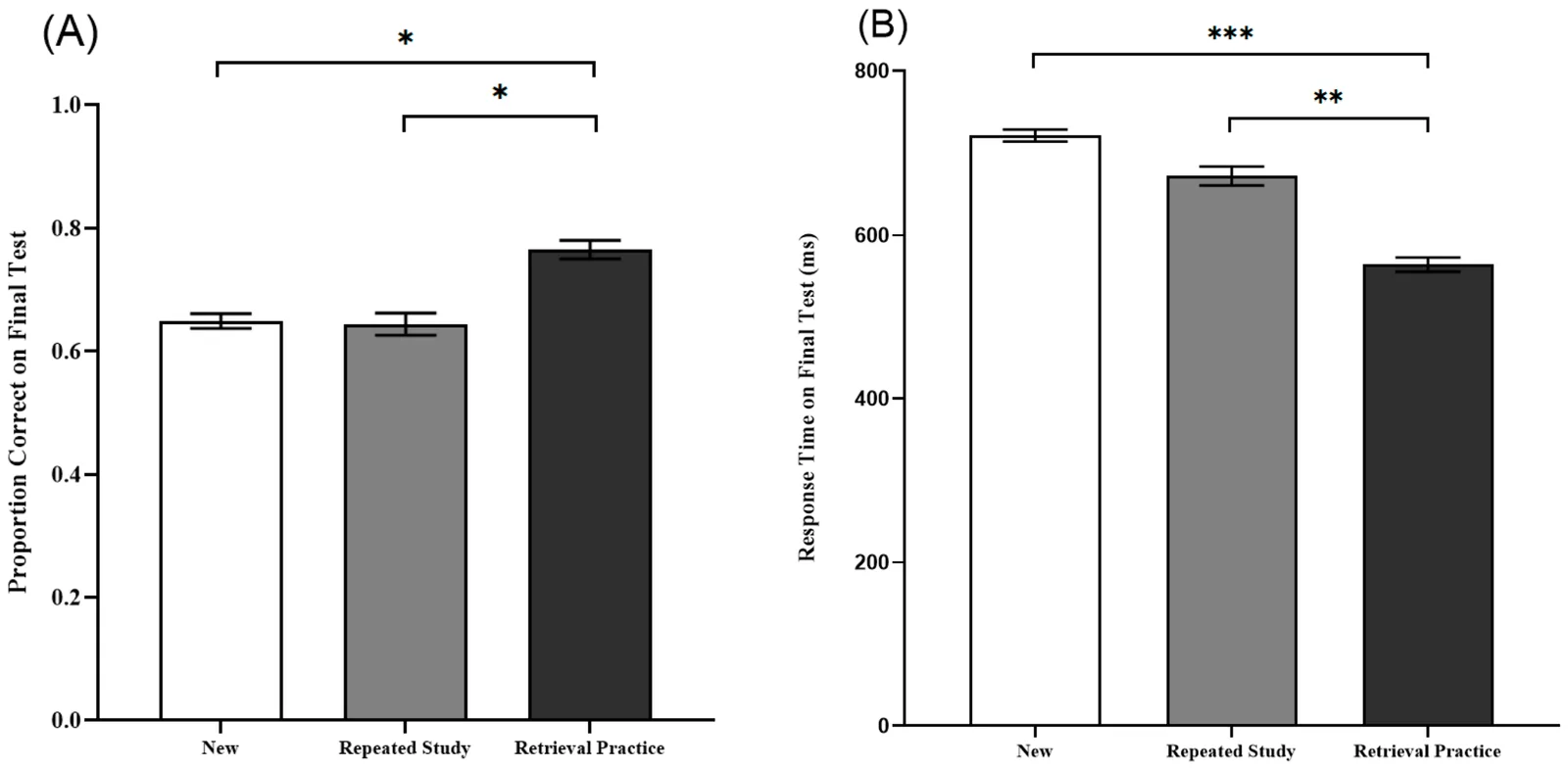
Results of the final recognition memory test. (**A**) Proportion of correct responses across the three learning conditions. (**B**) Response times across the three learning conditions. Error bars represent standard errors. Significance levels are indicated as follows: * *p* < 0.05, ** *p* < 0.01, and *** *p* < 0.001.

The analyses of Experiment 1 focused on performance in the final recognition memory test, as illustrated in Figure 2. Statistical analyses were performed in R (R Core Team, 2019), with mixed-effects models implemented using the lme4 package (Bates et al., 2015). The significance of fixed effects was assessed using likelihood ratio tests, and subsequent pairwise comparisons were conducted using the emmeans package (Lenth, 2018).

Recognition accuracy in the final memory test across the three conditions is presented in Figure 2A. Binary accuracy scores, coded as 1 for correct responses and 0 for incorrect responses, were analyzed using a mixed-effects logistic regression model (Jaeger, 2008). Learning condition (repeated study, retrieval practice, and new condition) was specified as the fixed effect. The model was implemented with the glmer function in the lme4 package (Bates et al., 2015) in R (R Core Team, 2019). The random-effects structure included by-participant random intercepts and slopes for learning condition, as well as by-item random intercepts. By-item random slopes for learning condition were not included because learning condition did not vary within items (Winter, 2019). A likelihood ratio test revealed a significant effect of learning condition (χ^2^(2) = 9.2053, *p* < 0.05). Post hoc comparisons with Tukey correction further revealed higher recognition accuracy in the retrieval practice condition than in both the new condition (Estimate = 0.5660, *SE* = 0.202, *z* = 2.805, *p* < 0.05) and the repeated study condition (Estimate = 0.6315, *SE* = 0.232, *z* = 2.721, *p* < 0.05). No significant difference was observed between the repeated study and new conditions (Estimate = 0.0655, *SE* = 0.202, *z* = 0.325, *p* = 0.9435).

Response times (RTs) in the final recognition memory test are presented in Figure 2B. The RT analysis was restricted to trials with correct responses, and observations falling beyond ±3 SD from the grand mean were excluded. RTs were log-transformed to reduce distributional skewness. The transformed RT data were analyzed using a linear mixed-effects regression model (Baayen et al., 2008), implemented with the lmer function in the lme4 package in R. Learning condition was modeled as a fixed effect, with random intercepts for participants and items and a by-participant random slope for learning condition. A likelihood ratio test revealed a significant effect of learning condition (χ^2^(2) = 25.364, *p* < 0.001). Post hoc comparisons with Tukey correction showed slower RTs in the new condition than in the retrieval practice condition (Estimate = 0.0711, *SE* = 0.0136, *t* = 5.221, *p* < 0.001), and faster RTs in the retrieval practice condition than in the repeated study condition (Estimate = −0.0592, *SE* = 0.0163, *t* = −3.638, *p* < 0.01). No significant difference was observed between the new and repeated study conditions (Estimate = 0.0119, *SE* = 0.0120, *t* = 0.985, *p* = 0.5875).

## 3. Experiment 2

### 3.1. Methods

#### 3.1.1. Participants

The participants were the same as those in Experiment 1.

#### 3.1.2. Materials

The same forty-eight French hyphenated compound words learned in Experiment 1 were used in Experiment 2.

#### 3.1.3. Design and Procedure

Experiment 2 was conducted 15 min after the completion of Experiment 1. Participants were instructed to identify and judge the stimuli presented on the screen using the rapid serial visual presentation (RSVP) paradigm. Participants were seated approximately 60 cm from a color monitor with a resolution of 1920 × 1080 pixels and a refresh rate of 60 Hz. Each trial began with a fixation cross (“+”) displayed for 1000 ms, followed by an RSVP stream. Each item in the stream, including targets and distractors, was presented for 100 ms without an inter-stimulus interval. The two morphemic components of a learned French compound word served as T1 and T2. T2 appeared either 300 ms after T1 at Lag 3 or 700 ms after T1 at Lag 7, with Lag 3 corresponding to the typical attentional blink time window. Thus, the Lag 3 condition contained two intervening distractors between T1 and T2, whereas the Lag 7 condition contained six intervening distractors. Distractors were digits from 2 to 9.

**Figure 3:**
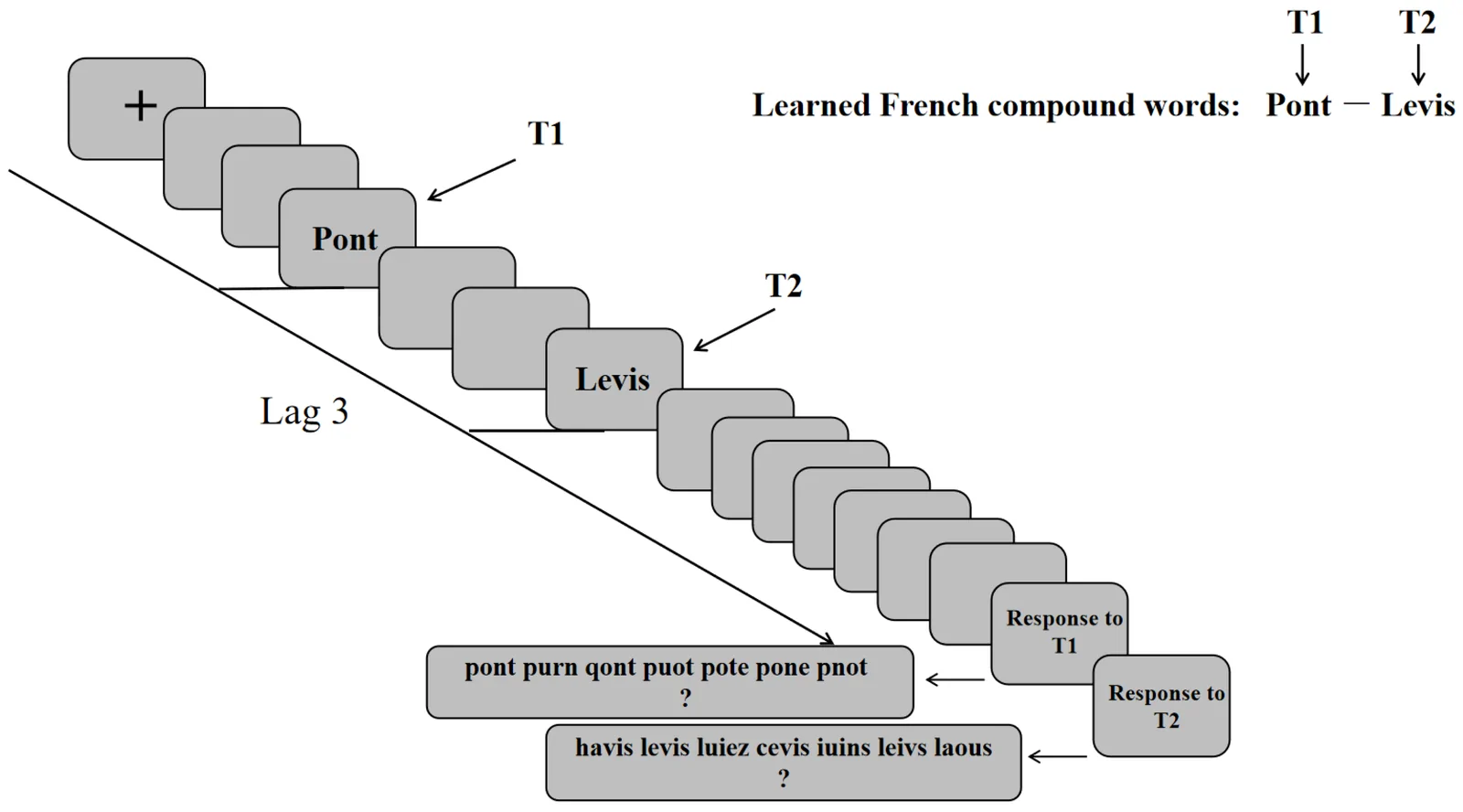
Schematic illustration of a trial sequence. T1 and T2 refer to the first and second targets, respectively. Lag denotes units of time between the two targets (Lag 3 = 300 ms). This scheme illustrates a Lag 3 trial, in which T2 appears 300 ms after T1, with two distractors between T1 and T2.

Following the RSVP stream, a response screen was presented with T1 and six distractor options. Participants identified the target by selecting the corresponding option with the mouse. Once the T1 response was completed, a second response screen with the same format was presented for T2 identification (see Figure 3). Participants were instructed to identify T1 and T2 in their order of presentation and to select the “?” option at the bottom of the screen if they were unable to identify the target.

The presented French words were randomly selected, and each item appeared only once, evenly distributed across the temporal lag conditions. In total, 48 French compound words were used; the left component of each compound word served as T1 (e.g., pont in pont-levis), and the right component as T2 (e.g., levis in pont-levis). The distractor options displayed on the response screens were orthographically similar to T1 or T2, thereby reducing the likelihood that participants could identify the targets based solely on their visual form.

Prior to the formal experiment, participants completed a five-trial practice block, and these trials were excluded from the formal analysis. After the practice block, each participant proceeded to the main task, which included 48 trials divided equally across four blocks. Each experimental block included both learning conditions (repeated study and retrieval practice) and one or both temporal lag conditions (Lag 3 and Lag 7). The presentation order of the French hyphenated compound words across the two learning conditions was counterbalanced.

#### 3.1.4. Results

**Figure 4:**
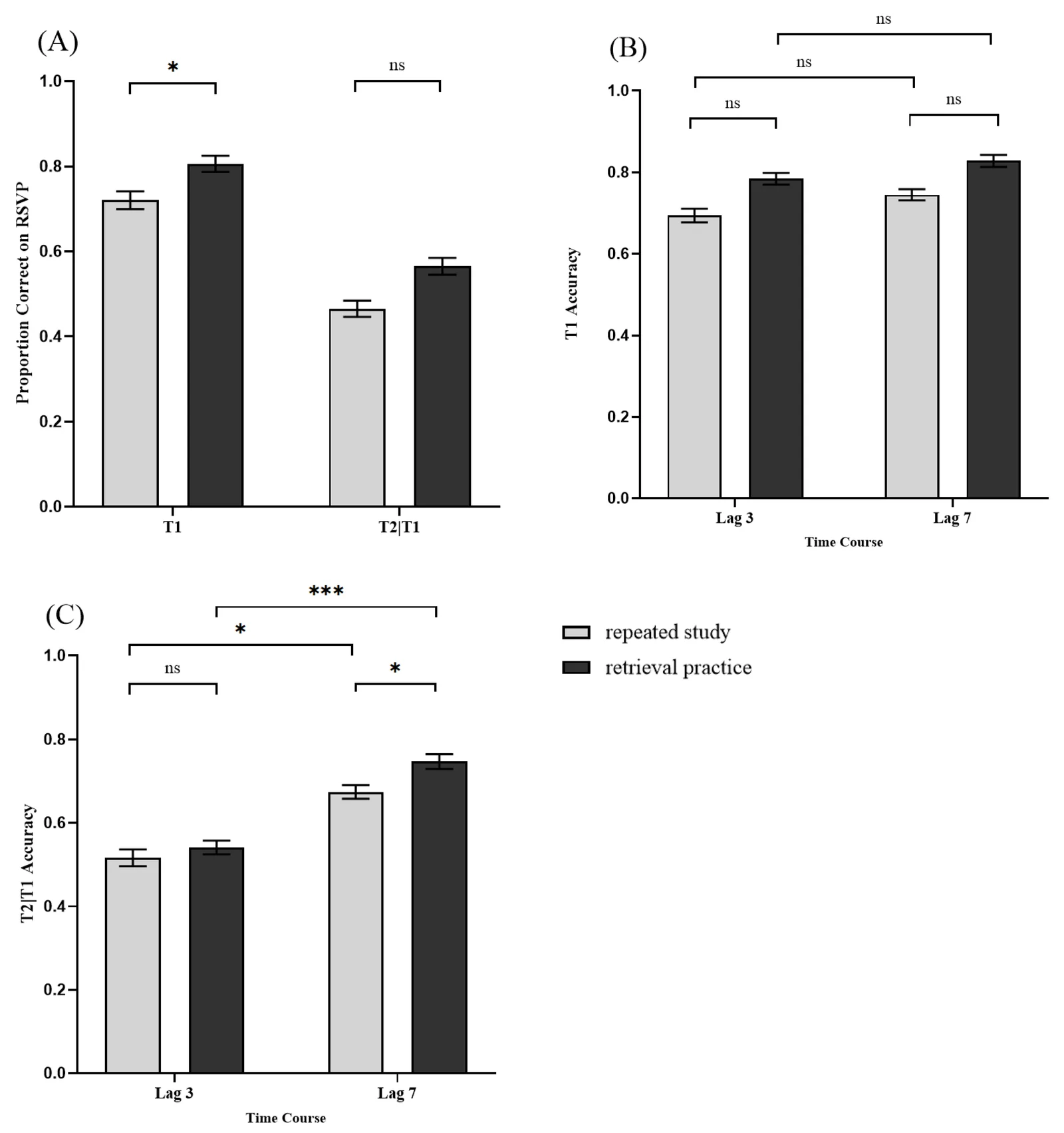
Accuracy results for T1 and T2|T1. (**A**) The overall T1 and T2|T1 accuracy under the two learning conditions. (**B**) The mean accuracy of T1 at Lag 3 and Lag 7 under the two learning conditions. (**C**) The mean accuracy of T2|T1 at Lag 3 and Lag 7 for the two learning conditions. Significance levels are indicated as follows: * *p* < 0.05, *** *p* < 0.001; ns, not significant.

Overall accuracy was calculated for T1 and T2|T1 under the retrieval practice and repeated study conditions, as shown in Figure 4A. Accuracy at Lag 3 and Lag 7 was also calculated separately for T1 (Figure 4B) and T2|T1 (Figure 4C) under the two learning conditions. Following Chun and Potter (1995), responses were scored as correct regardless of the order in which the two targets were identified.

For overall T1 accuracy, learning condition was modeled as a fixed effect, with by-participant random intercepts and slopes for learning condition and by-item random intercepts. A likelihood ratio test comparing models with and without the fixed effect revealed a significant effect of learning condition (χ^2^(1) = 5.4246, *p* < 0.05). The corresponding analysis for overall T2|T1 accuracy yielded no significant effect of learning condition (χ^2^(1) = 3.4353, *p* = 0.064).

For identification accuracy of T1 (Figure 4B), likelihood ratio tests showed a significant main effect of learning condition (χ^2^(1) = 5.649, *p* < 0.05), but no significant temporal lag effect (χ^2^(1) = 2.1275, *p* = 0.1447) or interaction between learning condition and temporal lag (χ^2^(1) = 0.0021, *p* = 0.9632). However, post hoc comparisons with Tukey correction showed that the differences between repeated study and retrieval practice were not significant at either Lag 3 (Estimate = 0.531, *SE* = 0.311, *z* = 1.708, *p* = 0.0876) or Lag 7 (Estimate = 0.551, *SE* = 0.314, *z* = 1.755, *p* = 0.0793).

For the accuracy of T2|T1, likelihood ratio tests showed main effects of learning condition (χ^2^(1) = 4.4202, *p* < 0.05) and temporal lag (χ^2^(1) = 12.648, *p* < 0.001), but no significant interaction between learning condition and temporal lag (χ^2^(1) = 1.0684, *p* = 0.3013). Post hoc comparisons with Tukey correction showed that the learning condition effect was significant at Lag 7 (Estimate = 0.736, *SE* = 0.324, *z* = 2.272, *p* < 0.05), but not at Lag 3 (Estimate = 0.261, *SE* = 0.323, *z* = 0.807, *p* = 0.4195).

The temporal lag effect was further examined separately for the retrieval practice and repeated study conditions. Post hoc comparisons with Tukey correction showed that the temporal lag effect was significant for both the repeated study condition (Estimate = −0.64, *SE* = 0.323, *z* = −1.980, *p* < 0.05) and the retrieval practice condition (Estimate = −1.12, *SE* = 0.324, *z* = −3.444, *p* < 0.001), suggesting the presence of an AB in both learning conditions.

## 4. Discussion

The present study investigated whether retrieval practice facilitates memory retention and the subsequent rapid identification of newly learned L3 French word components under RSVP conditions. Experiment 1 replicated the classical testing effect: French compound words learned through retrieval practice were recognized more accurately and more quickly than those learned through repeated study. Experiment 2 further showed that retrieval practice facilitated T2 identification in the RSVP task, with a significant advantage at Lag 7 and a numerical advantage at Lag 3. However, the interaction between learning condition and temporal lag was not significant. Therefore, the present findings are better interpreted as evidence that retrieval practice facilitates the rapid identification of newly learned word components, particularly when attentional resources are relatively recovered, rather than as strong evidence that it reliably reduces the magnitude of the attentional blink. Overall, the results suggest that retrieval practice strengthens newly learned lexico-semantic representations and makes them more accessible during subsequent rapid visual processing.

The findings of Experiment 1 are consistent with a large body of research on the testing effect, which shows that active retrieval enhances later memory more effectively than repeated exposure (Adesope et al., 2017; Huang et al., 2025; Karpicke & Roediger, 2008; Long et al., 2026; Roediger & Karpicke, 2006; Rowland, 2014). In the present study, retrieval practice led to higher recognition accuracy and faster response times in the final old/new recognition test. This pattern suggests that retrieval practice not only improved the availability of the newly learned French words, but also made access to these words more efficient. Compared with repeated study, retrieval practice required participants to actively reconstruct the French compound word from its Chinese cue and compare their response with corrective feedback, which likely strengthened the association between the Chinese translation and the French word form.

This interpretation is compatible with elaborative retrieval theory. According to this account, retrieval practice benefits memory because the act of retrieval activates information semantically related to the cue and integrates it with the retrieved target (Carpenter, 2009, 2011; Carpenter & Yeung, 2017). In the present study, the retrieval cue was the Chinese translation of the French compound word. Successful retrieval may therefore have promoted the integration of meaning, orthographic form, and compound structure. For semantically opaque French compound words, whose meanings cannot be fully inferred from their morphemic components, such integration may be especially important. The retrieval practice condition may have encouraged participants to form stronger links between the whole compound word, its Chinese meaning, and its internal morphemic components, providing a plausible explanation for the superior recognition performance observed in Experiment 1.

Experiment 2 extended these findings by examining the effects of retrieval practice in a rapid visual identification task. The results showed that T2 identification accuracy was higher for items learned through retrieval practice than for items learned through repeated study. This suggests that the effect of retrieval practice was not limited to the final recognition test, but also transferred to a task requiring rapid processing of briefly presented word components. Because T1 and T2 were morphemic components of the same learned compound word, successful T1 processing could have activated the newly formed lexical representation of the compound and increased the accessibility of T2. In this sense, retrieval practice may have facilitated the use of lexico-semantic associations during rapid visual word identification. It should be noted that Experiment 2 did not assess naturalistic recognition of familiar words; instead, it used an RSVP task requiring rapid identification of morphemic components from recently learned French compound words. Performance therefore likely reflected both rapid visual identification and episodic retrieval of newly formed lexical representations under temporal attentional constraints. Accordingly, the findings suggest that retrieval practice facilitated the identification of newly learned word components in the RSVP task, rather than naturalistic lexical recognition.

Importantly, the retrieval practice advantage was significant at Lag 7 but only numerical at Lag 3. This pattern should be interpreted with caution because the interaction between learning condition and temporal lag was not significant. Thus, the present findings do not provide strong statistical evidence that retrieval practice specifically reduced the magnitude of the attentional blink. A plausible account of this pattern is that retrieval practice strengthened the lexico-semantic representations of the newly learned compound words, but the use of these strengthened representations still depended on the availability of attentional resources. At Lag 3, T2 appeared while T1 was still undergoing attentional selection and working memory consolidation. Under this resource-limited condition, participants may have had insufficient time or cognitive resources to use the compound-level association activated by T1 to facilitate T2 identification. In other words, retrieval practice may have enhanced the quality of lexical representations, but it did not remove the temporal bottleneck imposed by T1 consolidation. At Lag 7, when attentional resources had relatively recovered, the strengthened lexico-semantic representations were more likely to support T2 identification, resulting in a significant retrieval practice advantage.

The significant temporal lag effect for T2|T1 accuracy confirmed the presence of an attentional blink in both learning conditions, with lower T2 identification accuracy at Lag 3 than at Lag 7. This pattern is consistent with classic accounts of the AB, which attribute impaired T2 identification to limitations in attentional allocation and working memory consolidation when two targets occur in close temporal proximity (Chun & Potter, 1995; Dux & Marois, 2009; Raymond et al., 1992; Vogel & Luck, 2002). Together with the non-significant Learning Condition × Lag interaction, this finding indicates that retrieval practice improved the accessibility of newly learned lexical representations without eliminating the temporal attentional constraints underlying the AB.

The present findings also speak to research showing that semantic or lexical relatedness between T1 and T2 can facilitate T2 identification in AB tasks (Cao et al., 2023; Potter et al., 2002). Previous studies have suggested that when T1 and T2 are semantically related, processing T1 may pre-activate information related to T2. The current study extends this line of research by showing that such facilitation may depend not only on pre-existing semantic relations, but also on how newly learned lexical associations are acquired. Unlike previous studies using familiar words or translation equivalents, the present study used newly learned L3 French compound words. The results suggest that retrieval practice may help learners build lexical associations that support later rapid identification, even though these associations did not significantly change the overall size of the attentional blink.

Theoretically, this study links retrieval-based learning with temporal attention by showing that the quality of lexical encoding can influence later performance in an RSVP task. Most previous studies on retrieval practice have focused on delayed memory tests, recognition performance, or neural correlates of vocabulary retention. The present findings extend this work by showing that retrieval practice can also facilitate the rapid identification of newly learned word components. At the same time, the non-significant interaction between learning condition and temporal lag indicates that strengthened lexical representations and temporal attentional constraints should not be treated as the same process. Retrieval practice appears to improve the accessibility of newly learned lexical information, whereas the attentional blink reflects a broader temporal bottleneck that remains present even when learned materials are used.

The findings also have implications for L3 vocabulary learning. Retrieval practice may be useful for learning semantically opaque compound words because it encourages learners to actively reconstruct the target word and integrate its form and meaning. Such practice may produce lexical representations that are not only better retained, but also more efficiently accessed during later processing. However, because the present task involved briefly presented word components in an RSVP paradigm, further research is needed to determine whether this benefit generalizes to more naturalistic reading or listening contexts.

Several limitations should be acknowledged. First, because Experiment 2 involved newly learned morphemic components presented in an RSVP paradigm, rather than familiar lexical items encountered in naturalistic contexts, the generalizability of the present findings to everyday word recognition remains limited. Second, although all participants were native Chinese speakers with English as their second language and reported no prior formal learning experience with French, we did not collect detailed information about their experience with other foreign languages. Such language-learning experience may influence the formation of new lexical representations and should be more carefully controlled in future research. Third, although the sample was relatively homogeneous in terms of native language and French-learning background, potential sources of heterogeneity remained, including variation in English proficiency, working memory capacity, and vocabulary-learning strategies. These factors may affect both vocabulary learning and rapid target identification in the RSVP task. Fourth, only two temporal lags were included: Lag 3 and Lag 7. Although these two lags allowed us to compare performance inside and outside the typical AB window, more lag conditions would provide a more fine-grained estimate of AB magnitude. Finally, the non-significant interaction between learning condition and temporal lag should be interpreted with caution. It remains possible that the present study was underpowered to detect an interaction of this magnitude. Therefore, the absence of a significant interaction should not be taken as definitive evidence that retrieval practice does not influence AB magnitude. Future studies with larger samples, larger item sets, and more fine-grained lag manipulations are needed to test this possibility more directly.

## 5. Conclusions

This study shows that retrieval practice enhances memory retention and facilitates the rapid identification of newly learned L3 French word components under RSVP conditions. The findings suggest that retrieval practice may strengthen lexico-semantic associations among the learned compound word, its Chinese meaning, and its morphemic components, thereby making the newly acquired lexical representations more accessible during subsequent RSVP processing. However, because the interaction between learning condition and temporal lag was not significant, the present results are better interpreted as evidence for a facilitative effect of retrieval practice on the rapid identification of newly learned word components, particularly when attentional resources are relatively recovered, rather than as direct evidence for a reliable reduction in the magnitude of the attentional blink. These findings bridge research on retrieval-based learning and temporal attention, highlighting the potential role of lexico-semantic access in both vocabulary retention and rapid visual word processing. For L3 learners, incorporating retrieval practice into vocabulary training may therefore help build more durable and accessible lexical representations.

## Data Availability

The datasets generated during and/or analyzed during the current study are available from the corresponding author upon reasonable request due to privacy.
